# Brain-Region-Specific Genes Form the Major Pathways Featuring Their Basic Functional Role: Their Implication in Animal Chronic Stress Model

**DOI:** 10.3390/ijms25052882

**Published:** 2024-03-01

**Authors:** Vladimir Babenko, Olga Redina, Dmitry Smagin, Irina Kovalenko, Anna Galyamina, Natalia Kudryavtseva

**Affiliations:** 1 Federal Research Center Institute of Cytology and Genetics, Siberian Branch of Russian Academy of Sciences, Novosibirsk 630090, Russia; oredina@bionet.nsc.ru (O.R.); smagin@bionet.nsc.ru (D.S.); koir@bionet.nsc.ru (I.K.); galyamina@bionet.nsc.ru (A.G.); natnik@bionet.nsc.ru (N.K.); 2 Pavlov Institute of Physiology, Russian Academy of Sciences, Saint Petersburg 199034, Russia

**Keywords:** animal chronic stress model, brain regions, dopamine, serotonin

## Abstract

The analysis of RNA-Sec data from murine bulk tissue samples taken from five brain regions associated with behavior and stress response was conducted. The focus was on the most contrasting brain region-specific genes (BRSG) sets in terms of their expression rates. These BRSGs are identified as genes with a distinct outlying (high) expression rate in a specific region compared to others used in the study. The analysis suggested that BRSG sets form non-randomly connected compact gene networks, which correspond to the major neuron-mediated functional processes or pathways in each brain region. The number of BRSGs and the connection rate were found to depend on the heterogeneity and coordinated firing rate of neuron types in each brain region. The most connected pathways, along with the highest BRSG number, were observed in the Striatum, referred to as Medium Spiny Neurons (MSNs), which make up 95% of neurons and exhibit synchronous firing upon dopamine influx. However, the Ventral Tegmental Area/Medial Raphe Nucleus (VTA/MRN) regions, although primarily composed of monoaminergic neurons, do not fire synchronously, leading to a smaller BRSG number. The Hippocampus (HPC) region, on the other hand, displays significant neuronal heterogeneity, with glutamatergic neurons being the most numerous and synchronized. Interestingly, the two monoaminergic regions involved in the study displayed a common BRSG subnetwork architecture, emphasizing their proximity in terms of axonal throughput specifics and high-energy metabolism rates. This finding suggests the concerted evolution of monoaminergic neurons, leading to unique adaptations at the genic repertoire scale. With BRSG sets, we were able to highlight the contrasting features of the three groups: control, depressive, and aggressive mice in the animal chronic stress model. Specifically, we observed a decrease in serotonergic turnover in both the depressed and aggressive groups, while dopaminergic emission was high in both groups. There was also a notable absence of dopaminoceptive receptors on the postsynaptic membranes in the striatum in the depressed group. Additionally, we confirmed that neurogenesis BRSGs are specific to HPC, with the aggressive group showing attenuated neurogenesis rates compared to the control/depressive groups. We also confirmed that immune-competent cells like microglia and astrocytes play a crucial role in depressed phenotypes, including mitophagy-related gene *Prkcd*. Based on this analysis, we propose the use of BRSG sets as a suitable framework for evaluating case–control group-wise assessments of specific brain region gene pathway responses.

## 1. Introduction

The advent of the RNA sequencing method, known as RNA-seq, ushered in a new era in molecular biology and genetics [1]. This technique allows for the simultaneous measurement of all genes in the genome, thereby facilitating a comprehensive transcriptome-wide analysis. The data obtained from this method have enabled researchers to reconstruct gene networks through gene expression covariance analysis, thereby validating or updating existing knowledge about gene networks. Moreover, the subsequent years have seen a continual expansion of these data.

Intensive research on brain regions and cell-specific gene expression, as well as the elucidation of single-cell taxonomy using the RNA-Seq protocol, commenced in 2014 [2,3,4,5,6]. A definitive comparative paper on six basic cell types (Astrocyte, Neuron, Oligodendrocyte, Oligodendrocyte Precursor, Microglia, and Endothelial Cells) identified cell-specific genes [7]. Alongside the basic brain cell types, the single-cell RNA (scRNA) data offered valuable insights into cell types developmental transition stages and taxonomy in various brain regions.

Research on neural cell lines highlights the heterogeneity of cell-specific expression profiles. Another crucial task is identifying brain region-specific markers. A recent paper on this topic [8] utilized GTEX expression data to clarify five brain regions, specifically gene sets determined by a deep learning clustering procedure. However, there are still numerous brain sub-regions that are not included in this list.

The neuron, characterized by its active genes ensembles, is the most varied cell type in the brain. Neuron types are predominantly categorized by the neurotransmitters they emit (glutamatergic, GABAergic, serotonergic, dopaminergic, etc.), which are accompanied by corresponding intracellular signaling pathways (cAMP/cGMP-mediated cascades, etc.). Neurons can also be classified by morphology and the electrophysiological and spike pattern properties of neuron types. For instance, over 200 neuron types have been reported in the hippocampal brain region, as detailed in the Hippocampome database [9,10].

The current study utilizes the data of the sensory contact model, which was later renamed to the chronic social conflict model [11,12,13]. This model enables the formation of alternative types of social behaviors in male mice. Specifically, it allows for the creation of “winners”, who result from repeated experiences of aggression accompanied by victories, and “losers”, who are formed through repeated experiences of defeats accompanied by chronic social stress.

As a result of prolonged positive or negative social experiences in 20 daily agonistic interactions, symptoms of pathological states begin to develop in male mice. These symptoms include a psychosis-like state, accompanied by signs of addiction, in the aggressive winners. Conversely, in the chronically defeated mice, a mixed anxiety/depression-like state is observed.

Five brain regions employed in the study play a significant role in the chronic stress response and behavior are the Hypothalamus (HPT), Hippocampus (HPC), Dorsal Striatum (STR), Ventral Tegmental Area (VTA), and Midbrain Raphe Nuclei (MRN) [11]. These regions served as the foundation for elucidating Brain Region Specific Genes (BRSG) and their specific networks, similar to those described in [8].

The findings suggest that BRSGs are non-randomly enriched in connectivity, forming gene networks that demonstrate specific functions within the brain regions. This allowed us to annotate the brain regions based on their primary function supported by specific signaling gene pathway(s). It is worth noting that the majority of BRS genes are intrinsically linked to neuronal genes, as the pathways of glial cell types (with the exception of astrocytes to a certain extent) are relatively similar in expression rate across the brain regions considered.

## 2. Results

### 2.1. Major Neural Transmitters Glutamate vs. GABA Vesicular Transporters Expression in the Brain Regions

First, we deemed it reasonable to introduce the brain regions by profiling major outgoing neurotransmitters underlining the major transmission types of the five brain regions considered.

**Figure 1:**
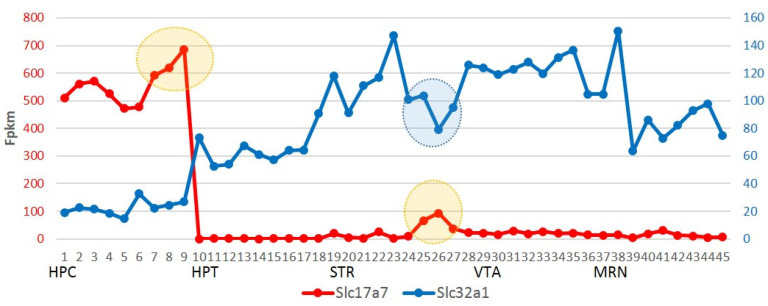
Depiction of glutamate VgluT1 (Slc17a7) and GABA VGAT (Slc32a1) vesicle transporters FPKM values across 45 samples of 5 brain regions (X axis); 9 samples (dots) per region consequently ordered as 3 controls, 3 aggressive (‘winners’), 3 depressive (‘losers’) mice. Encircled are three loser samples in HPC and STR regions discussed further.

To assess the neuronal signaling in relation to the major neurotransmitters (glutamate, GABA) and in this way roughly representing the brain regions, we plot the profile of GABA and glutamate vesicular transporters expression rate in 45-fold samples across 5 brain regions based on RNA-Seq data in Figure 1.

According to Figure 1, four regions outline the GABAergic efferent, with the hippocampus manifesting glutamatergic efferent, as previously reported elsewhere. We also observe the specific elevation of glutamate neurotransmitters expression rate in losers’ hippocampus and dorsal striatum regions (orange circles) while GABAergic emission in Medium Spiny Neurons (MSN) of striatum was correspondingly lowered (blue circle). Notably, there is an observation that GABAergic neurons emission is modulated with opiodergic signaling, reported in a recent survey [14], in particular in VTA afferent.

Aside from the two major neurotransmitters, we will annotate brain-region-specific ones below in the course of this study.

### 2.2. Selection of the Brain-Region-Specific (BRS) Genes Algorithm

We used the tissue specific index (TSI) for the selection of BRSGs reported in [15]:

$$TSI\;=\;x_{\max}/\sum x_{i},\;i\;=\;1,\ldots 5,$$

where *x* is an average expression rate (FPKM) in a certain brain region. We used a soft threshold of TSI > 0.5 for obtaining the BRSGs’ sample. For common BRS genes (VTA/MRN only), *x_max_ = x_vta_ + x_mrn_.*

**Figure 2:**
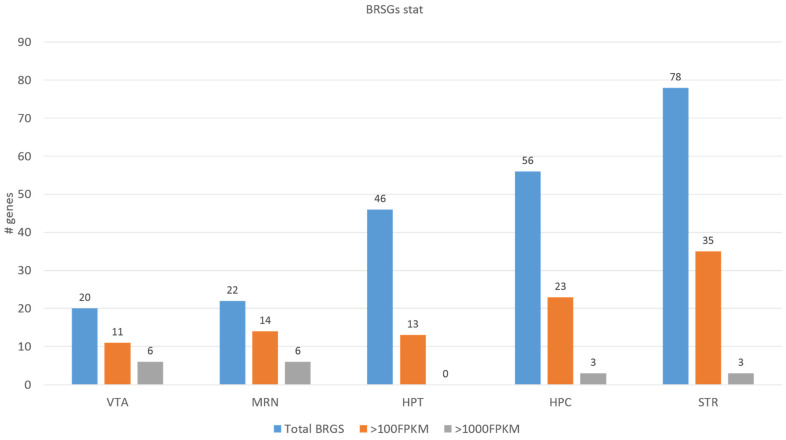
Distribution of BRSGs across 5 brain regions and their expression rate breakdown.

As a result, we ascertained 205 distinct genes in 5 regions, presented in Table S1. Table 1 shows the breakdown of BRSGs expression rate by brain regions based on Table S1, also highlighted by Figure 2 histogram.

Based on the data in Table 1, we may state that the most BRSGs were found in the STR region followed by HPC, HPT, MRN, and VTA. Conversely, the top number of highly expressed genes (>1000 FPKM) emerged in the VTA/MRN brain regions.

We report some of gene families expanded in BRSGs sets (see Table S1). We observed a subfamily of 4 phosphodiesterases forming a close network in the STR (*Pde10a*, *Pde1b*, *Pde2a*, *Pde7b*; GO:mmu00230) and performing purine metabolism in cAMP- and cGMP-mediated cascades, accompanied with the family of Voltage-gated potassium channel activity genes network (*Kcnh4*, *Kcnh3*, *Kcnip2*, *Kcnj4*; GO:0005249), and supplemented with *Ras*- family genes (*Rasd2*, *Rasgef1b*, *Rasgrp2*). *Drd1* and *Drd2* genes are specific for STR.

There was not any gene family wise enrichment in the HPT region (Table S1), while HPC may be marked with Neurogenic differentiation (NeuroD) factor genes network (*Neurod2*, *Neurod6)* and two *Corpus callosum* development genes network, *Rtn4r* and *Rtn4rl*2. Both VTA and MRN BRSGs feature neurofilament family genes network (*Nefl*, *Nefh*, *Nefm*) along with glycinergic transporters set (*Slc6a5*, *Slc6a9*).

Further, we assessed the BRSG content by analyzing the BRSG networks in each brain region.

### 2.3. Analysis of the Function (Gene Ontology) of the BRS Genes in Five Regions

We used the string-db.org service (accessed on 1 January 2024) to annotate the BRSG sets for each region, listed in Table S1, and have presented/analyzed sampled GO annotations/BRSGs subsets in the next sections. Full BRSG GO annotations in 5 regions are located in Tables S2–S6.

Due to four distinct confidence PPI layers of protein association rate in the string-db.org database, we listed the exhaustive stats for the brain regions for each of them in Table 2, stressing that nonrandom edges enrichment is present in each of the layer for each brain region.

The confidence score is ascertained through various sources/layers, including: (1) Co-occurrence Across Genomes, (2) Co-Expression, (3) Experimental/Biochemical Data, (4) Association in Curated Databases, (5) Co-Mentioned in Pubmed Abstracts. The highest level requires all layers confirmed.

As seen in Table 2, the vast majority of BRSGs within brain regions are interconnected at the low confidence level (at least one confidence layer). Considering medium level score (our accepted one), STR maintains the highest rate of edges per node density (2.8; Table 2) followed by HPT (2.4), while HPC (1.8) and MRN/VTA (1.3) maintained it the least due to functional/neuronal heterogeneity in HPC and small BRSG number in MRN/VTA.

Further on, we used a high confidence score (0.7) layer profile for displaying the dense pathways, given that it yields the core networks with clearer ability presenting them. Full GO annotation in the supplements were based on medium confidence score.

#### 2.3.1. Annotation of the BRSG Set of the Dorsal Striatum (STR)

**Figure 3:**
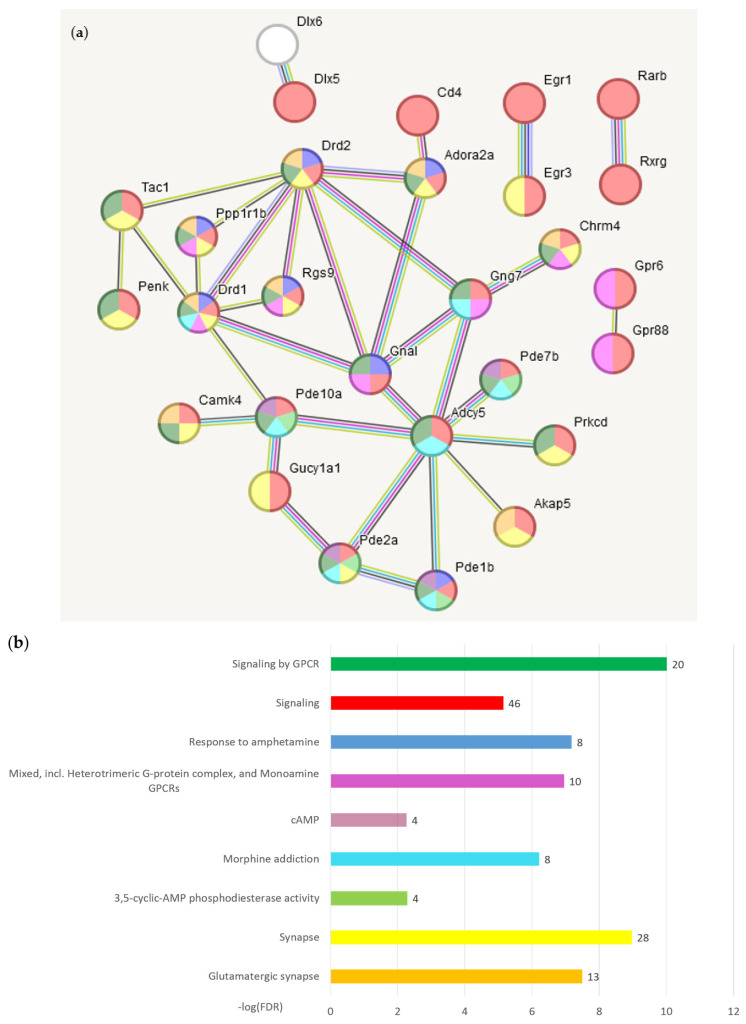
(**a**) 33 from 78 (singletons hidden) nodes gene network built from the BRSG list for STR (Table S1) with stringent links (high confidence score > 0.7; (string-db.org; Table 2). In total, 45 disconnected nodes (singletons) are hidden. (**b**) Statistics of selected categories of gene ontology. GO categories bar colors correspond to those of nodes shown in (**a**). The numbers of the corresponding genes are presented as bar labels. Full GO annotation presented in Table S2.

The gene network comprising 78 BRSGs in the STR region (Table 1), based on previous experimental and other data used in string-db.org, is shown in Figure 3.

**Figure 4:**
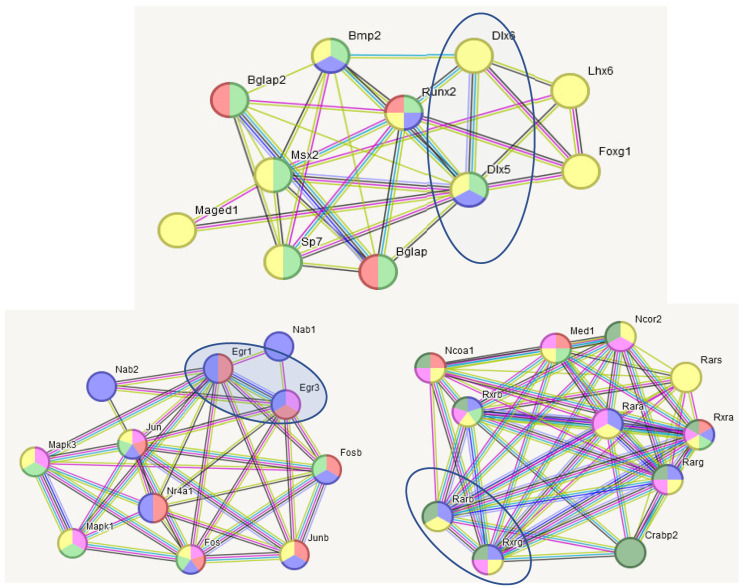
Three pairs of BRSGs from Figure 3 expanded to pathways belong to nuclear compartment-regulating transcription/deacetylation: *Dtx5–Dtx6*, *Egr1–Egr3*, *Rarb-Rxrg* (encircled). Color encoding is presented in Table 3.

We also expanded annotation of three connected gene pairs in Figure 3a, seeding them in string-db.org for connected networks (Figure 4; Table 3). *Gpr6-Gpr88* genes are membrane Gpcrs associated with Dopamine receptors in the cAMP cycle, as was annotated in GO (Table S1).

#### 2.3.2. BRSGs Projection against 9 STR Samples of Social Stress Model and Prkcd BRSG

The high interconnection rate implies the high co-variation of gene expression rate. We assessed co-variation based on STR, nine observations for each gene; when assessing gene clustering using the agglomerative hierarchical clustering (AHC) method, 71 out of 78 BRS genes fell into a single cluster (Table S3).

**Figure 5:**
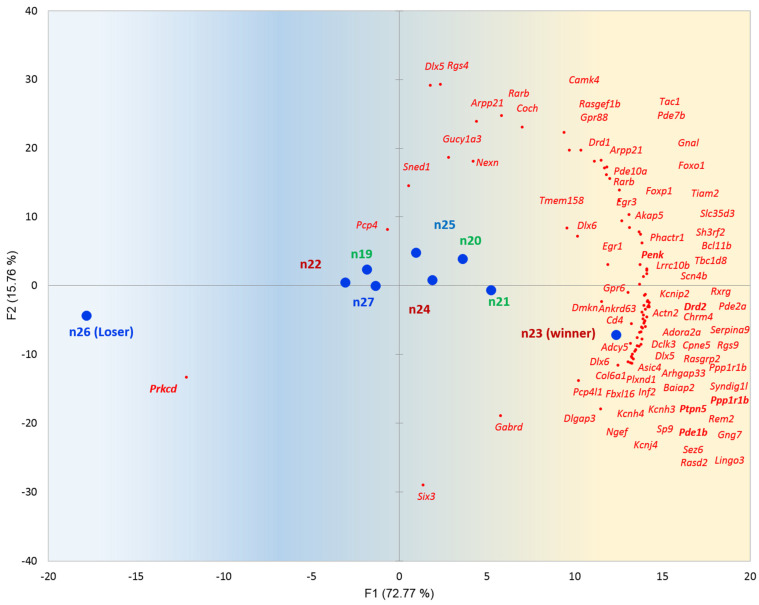
PCA plot of 78 STR BRS gene distribution (red dots; Table S1) against 9 samples (blue dots). Control samples are green shaded; aggressive (winners) have red tags; losers bear blue tags. Gradient color shading underlines the elevation of overall gene expression rate in the right part (blue → yellow).

The major secondary pathway of the STR region, consisting mostly of Medium Spiny Neurons (MSN), is the dopamine-induced cAMP signaling cascade [16], modulating the most of secondary networks in the STR upon chronic stress, as outlined in [17]. We replicated STR BRSGs’ set projection against the nine STR samples, presented in Figure 5.

Figure 5 features mostly *Drd1/Drd2*-mediated cAMP cascade BRS genes coordinated dynamics (76 on the right side of the Figure 5 plot). Many cAMP-specific genes fall in the STR BRSG pool (see Supplementary Table S1). Other than long known ones like *Drd1*, *Camk4*, *Drd2*, *Ppp1r1b*, *Pde10a*, *Adora2a*, *Adcy5*, *Ptpn5*, *Gpr88*, etc., *Lrrk2* kinase BRS gene is characterized quite recently [18] as one modulating D1 receptor signaling, along with many other BRSGs.

Also, we can see elevated expression of Protein Kinase C delta (*Prkcd*) in loser species (Figure 5, n26, n27) met with attenuation of dopamine-mediated cAMP signaling cascade [13]. It was mentioned manifesting a fear-related syndrome in anxious/depressive species since 2001 and later [19,20,21,22].

Recently, the confirmation of *Prkcd* localization in mitochondria and its involvement in *Prkn*-independent mitophagy [23,24] unveiled its possible role in a depressive disorder [25,26]. While *Prkcd* exemplifies a mitophagy performed by microglia/immune-competent cells (Munson et al., 2021, 2022) [23,24], it confirms playing a distinct role in the brain, being a striatum/amygdale-specific marker in a range of previous studies on MDD [19,20]. It is characteristic of multiple psychiatric statuses, including Early-Life Anxious Temperament [21,22] and depression/suicide behavior [27]. It is also observed expressing in specific *Prkcd*-positive GABAergic neurons within the central amygdala, and is shown to modulate/be modulated, in particular, by the Tissue plasminogen activator gene (*tpA*; *Plat*; [28]) affecting behavioral pattern. Bed nucleus of stria terminals (BNST) is also noted for stress-mediated *Prkcd* expression impact [29], assuming that inflammation-mediated mitophagy may be the cause.

Notably, we maintained only a single mouse with a distinct manifestation of *Prkcd* expression outburst while abrogating the dopamine influx (Figure 5), implying its non-compulsory role in a depressive phenotype, though significantly jeopardizing it (Figure 5).

**Figure 6:**
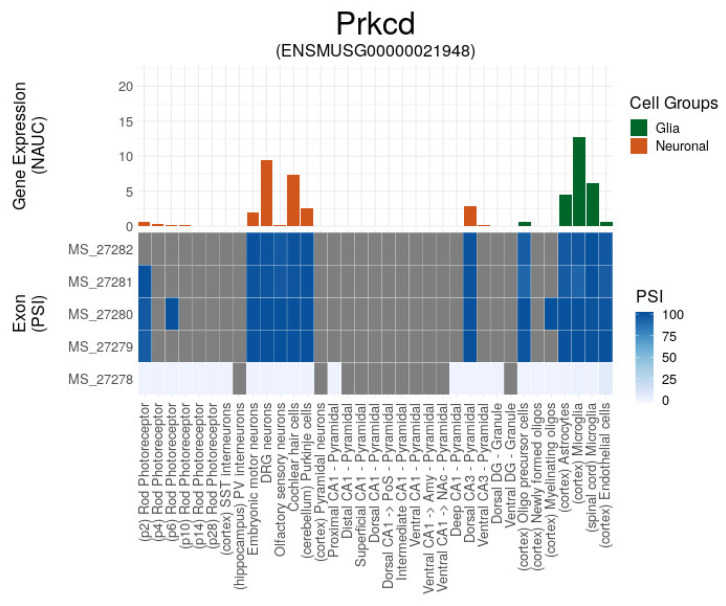
*Prkcd* expression in brain cells (Ascot database; [30]). It maintains at least five cassette exons, which feature majorly switch-on/off expression mode according to PSI score. It expresses in microglia (mitochondria) and in DRG neurons/cochlear hair cells.

It is also still not clear whether the *Prkcd* augmentation effect is provided by glial or neuronal cells, since microglial *Prkcd* expression is the highest one, according to the mouse brain expression atlas (Figure 6).

#### 2.3.3. Mitophagy Specifics in Social Stress Model

**Figure 7:**
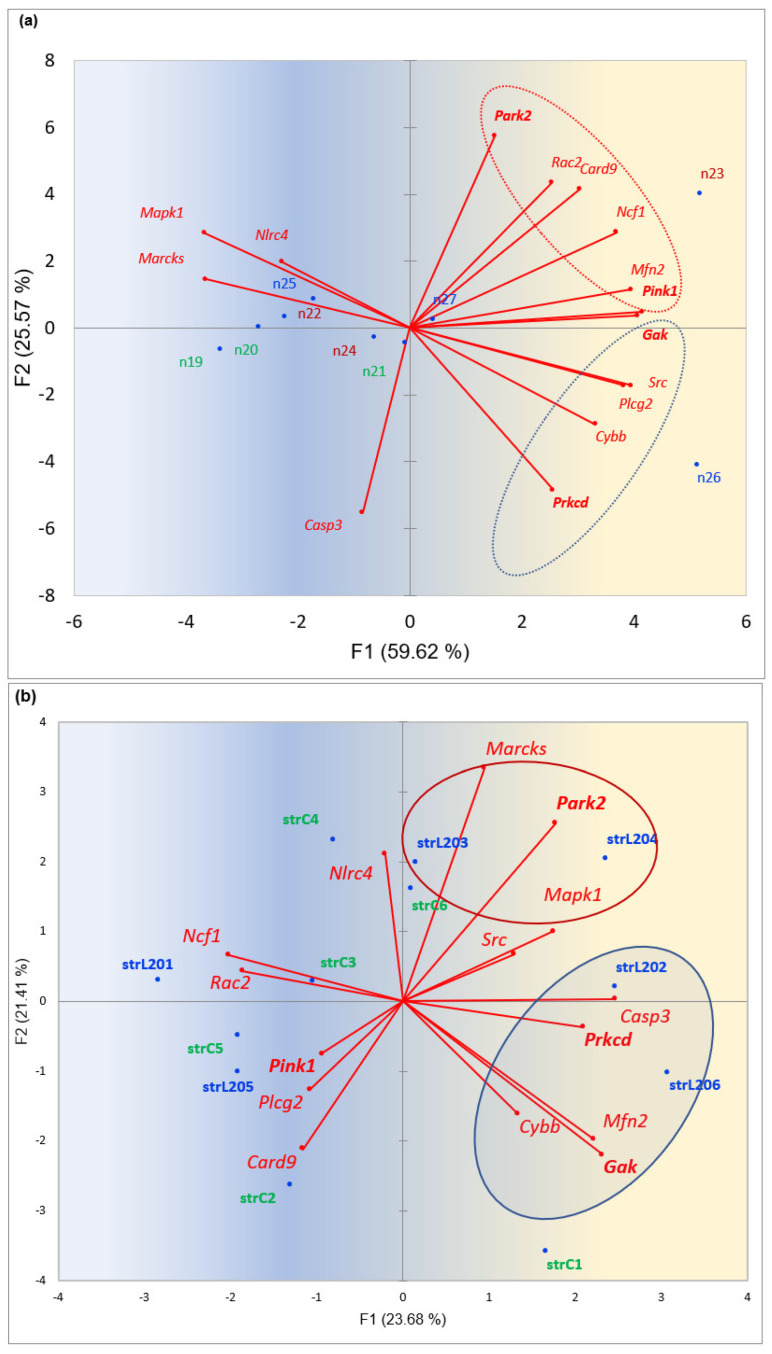
(**a**) PCA plot of mitophagy-related genes (mmu04137 (mitophagy); GO:0033554 (Cellular response to stress); GO:0048518 (Positive regulation of biological process)); 15 genes, including *Prkcd)* against 9 STR samples, 3 groups: control (green shaded), aggressive (red shaded), loser (blue shaded). Encircled are canonical (red circle) and PRKN-independent (blue circle) mitophagy cycles. Marker genes are in bold. Gradient color shading underlines the elevation of overall gene expression rate in the right part (blue → yellow). (**b**) PCA plot of 6 control mice (green) and 6 depressive mice (blue) against 15 mitophagy-related genes in STR (see (**a**)). Blue dots correspond to samples. Data from [17]. Encircled are the *Prkn*-independent mitophagy pathway (blue shaded) and the canonical *Prkn*-related one (red shaded).

As a mitophagy is likely a feature of MDD [25,26], we tested the distribution of mitophagy-related genes against the social stress groups, presented in Figure 7.

Figure 7a features two samples (aggressive, n23, depressive, n26) with distinct elevated mitophagy cycles. The aggressive one (n23) features a canonical mitophagy cycle based on *Pink1-Parkn* (*Park2*) tandem, while the loser species (n26) features a *Prkn*-independent mitophagy cycle based on the *Gak-Prkcd* interaction reported quite recently [23,24].

Based on Figure 7a,b, we may state that mitophagy networks vary both in genes content as well as in mitophagy type. Notably, StrL202 and StrL206 samples are the most dopamine-deficient ones, according to the data used [17], similar to n26 (Figure 7a).

#### 2.3.4. Connectome and GO Annotation for Hypothalamus (HPT) BRSGs Based on String-db.org Resource

**Figure 8:**
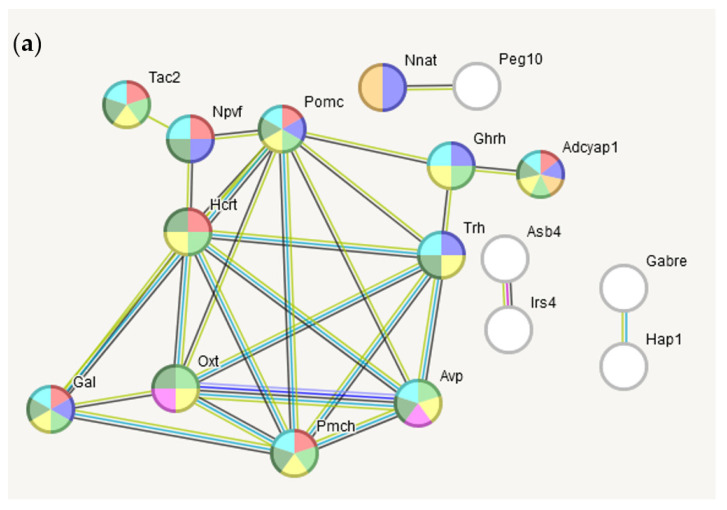

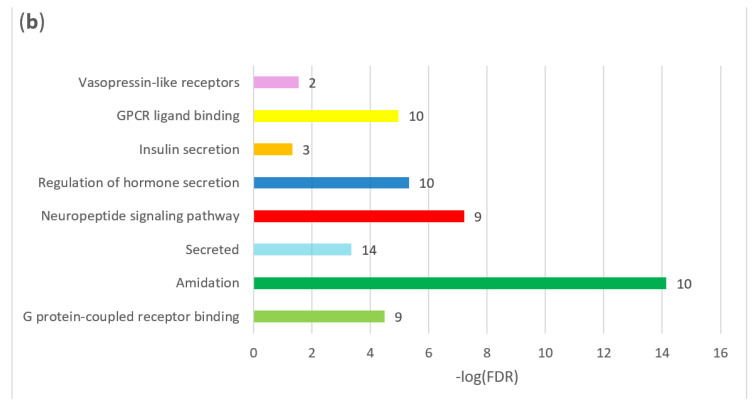
(**a**) Genes network of 17 from 46 (singletons hidden) nodes built with BRSG list for HPT (Table S1) with high confidence score (> 0.7; Table 2). The nodes’ color indicate GO categories shown in the chart below. (**b**) Statistics of selected categories of gene ontology (full GO ontology is provided in Table S4). The numbers of the corresponding genes are given as labels for each category. The statistic for the edges enrichment: observed edges number: 18; expected: 1 *p* < 1 × 10^−16^.

The gene network built on a set of 46 HPT BRSGs, confirmed experimentally (string-db.org), is presented in Figure 8.

**Figure 9:**
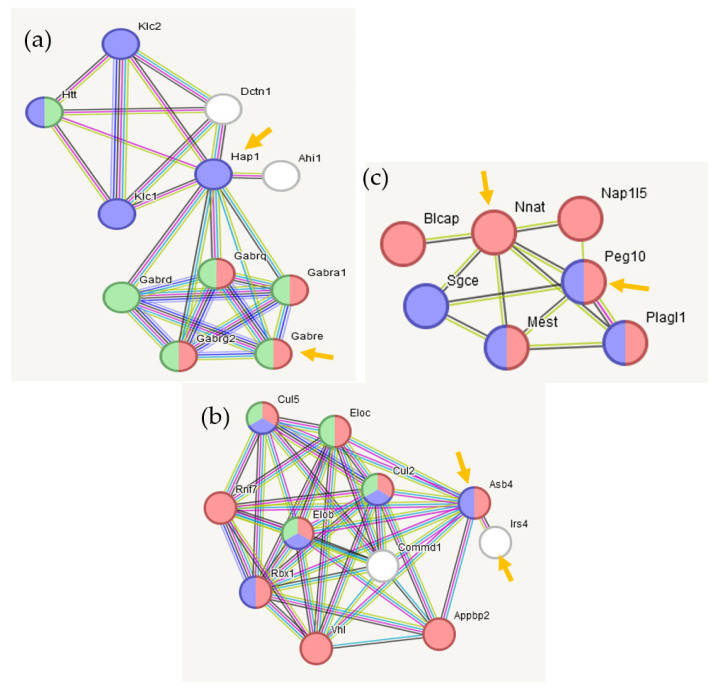
Three non-annotated BRSG pairs in Figure 8a expanded networks (by string-db.org). (**a**) *Gabre-Hap1*- pair extension; (**b**) *Asb4-Irs4* pair extension; (**c**) *Nnat*-*Peg10* pair extension. Seeding BRSGs are marked with orange arrows. GO color encoding for each genes network is in Table 4.

Commenting on Figure 9/Table 4, we note that hypothalamic *Irs4*-expressing neurons are involved in energy homeostasis (https://www.nature.com/articles/s41598-020-62468-z, accessed on 1 January 2024). As for *Nnat* (Neuronatin) -*Peg10* (retrosposon-derived Paternally-expressed gene), while the *Nnat* insulin secretion protein also regulates whole-body metabolism, we cannot find any confident annotation of its functional interaction evidence with the *Peg10*-encoding Gag-protein, other than the co-mentioning rate of the genes’ pair in the publications, noting that *Peg10* is involved in genetic imprinting by methylation (MP:0003121; 8 genes).

#### 2.3.5. BRSGs Projection against 9 HPT Samples of Social Stress Model

**Figure 10:**
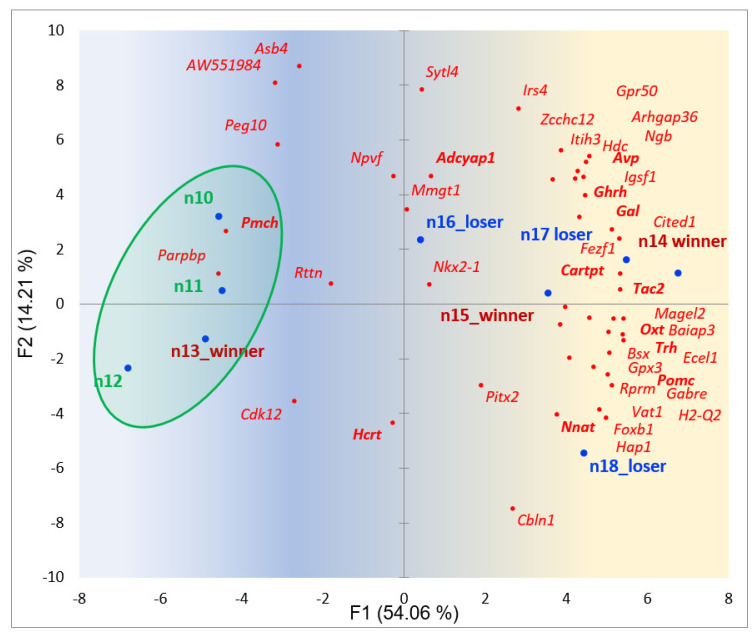
Distribution of 46 BRSGs (red dots) in the projection of 9 observations (blue dots) in the HPT region (n10, n11, n12–control (green shaded); n13, n14, n15–aggressive (red tags); n16, 17, n18–losers (blue tags)). Hormone-associated genes are bold typed. Gradient color shading underlines the elevation of the gene expression rate in the right part. Random clustering of affected subjects (5 samples) on the right side, rejected with *p*-value < 0.012 (binomial test).

The observed enriched number of edges (18; Figure 8) implies a strong genes’ covariance, confirmed in our PCA plot (Figure 10); the most BRS genes reside at the right part of the plot.

From Figure 10, it becomes obvious that affective individuals (winners, losers) experience a stress-followed hormonal/neuropeptide augmentation compared to the control group: only 9 genes are located on the left side of the graph, and 37 ones on the right. The uneven distribution of BRS genes across the left and right parts of the graph is confirmed by the random probability *p*-value < 0.00045 (binomial test), implying winner and loser groups’ non-random elevation of hormone-specific genes with high significance compared to controls. Hormone-related genes (GO:0005179) are: *Trh*, *Cartpt*, *Oxt*, *Ghrh*, *Avp*, *Pmch*, *Hcrt*, *Adcyap1*, *Pomc*, and *Gal*.

#### 2.3.6. GO Annotation of BRSGs in Hippocampus

**Figure 11:**
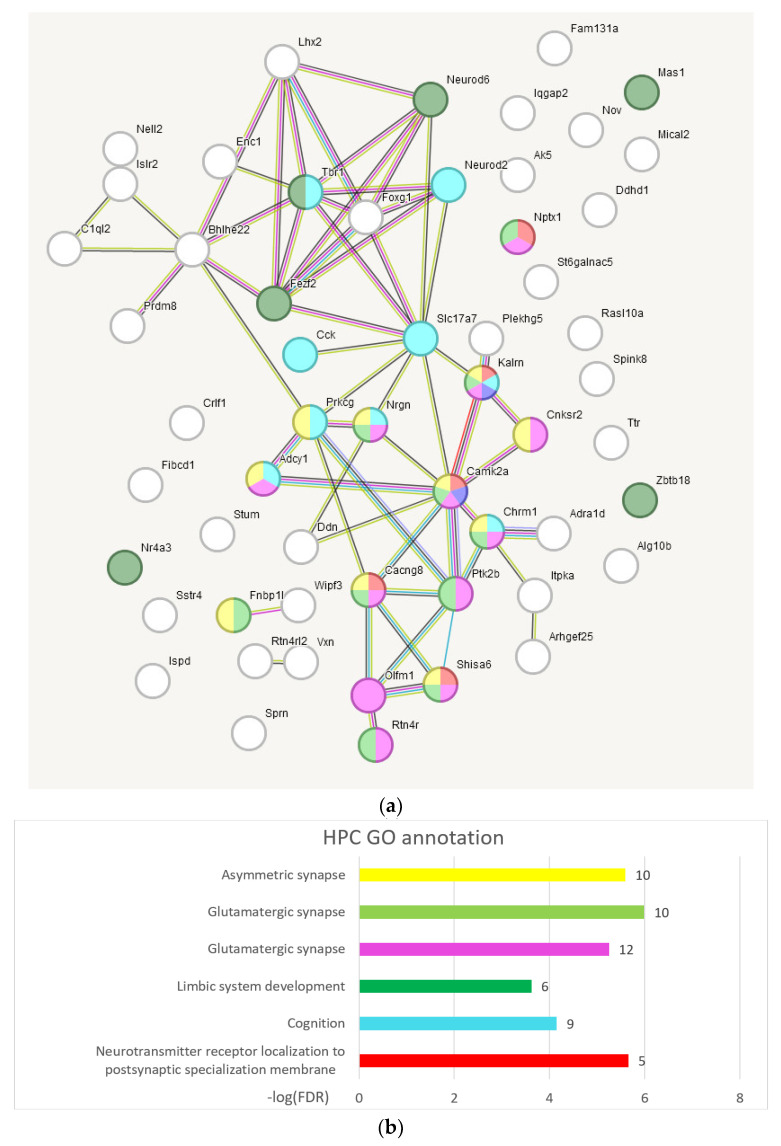
(**a**) Gene network of the 56 genes of the HPC BRSGs list using string-db.org. The colors indicate GO categories shown in the line chart below. (**b**) Statistics of selected categories of gene ontology. The number of the corresponding genes are given as bar labels for each GO category. The number of edges (59) exceeds the expected value (6) with probability *p* < 1 × 10^−16^ (string-db.org report). Full GO annotation is located in Table S5.

From Figure 11, we may conclude that the Glutamatergic synapse genes set is the most featured in HPC, along with neural developments genes. Agglomerative clustering featured the major cluster (29 nodes, Table S7, cluster 3), GO annotated as ‘Nervous System development’ (GO:0007399; 16 genes) and ‘Glutamatergic synapse’ (GO:0098978; 7 genes; see Table S7, the plot at the end of the list).

**Figure 12:**
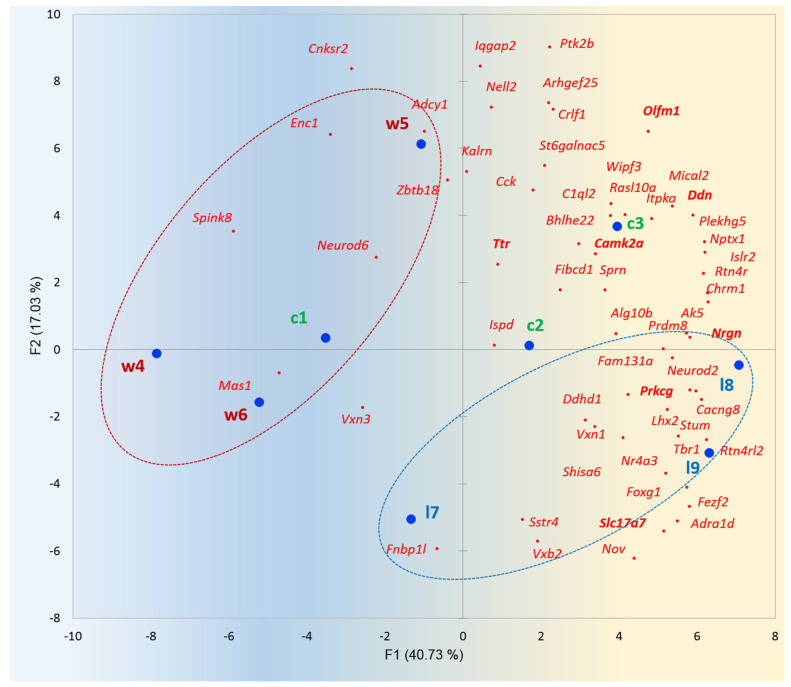
Distribution of 56 HPC BRSGs (red dots) in the projection of 9 observations (blue dots) in HPC region (c1, c2, c3–control (green tags); w4, w5, w6 –aggressive (red tags); l7, l8, l9–losers (blue tags)). Encircled are winner (red) and loser (blue) mice groups. Gradient color shading underlines the elevation of the gene expression rate in the right part. Random clustering of winners on the left side/losers on the right side, rejected with *p*-value < 0.125 (binomial test).

We were also interested in sample projection against BRS genes in HPC underlined by PCA plots for 9 HPC samples, presented in Figure 12.

Based on the group clustering in Figure 12, we may report that winners (aggressive) mice (w4–w6) maintain rather attenuated BRS genes expression in the hippocampus, implying lowed neural activity and glutamatergic synapse transmission intensity (Figure 12), while loser mice (l7–l9) display augmented activity in this region, including glutamatergic elevation according to the *Slc17a7* expression gradient. At the same time, it was reported that *Prkcg/Nrgn* elevation (Figure 12, bold typed) augments spatial learning and memory, as reported in [31]. These BRSGs feature loser mice cluster (blue-circled in Figure 12) manifesting increased hippocampus activity, and is additionally supported by the glutamatergic increase (yellow shaded area) shown in Figure 1. Thus, given a high co-variation with other hippocampal BRSGs, we may consider *Slc17a7* as a distinct driver gene and a molecular marker of HPC region expression dynamics.

#### 2.3.7. Neurogenesis in Social Stress Model Groups

**Figure 13:**
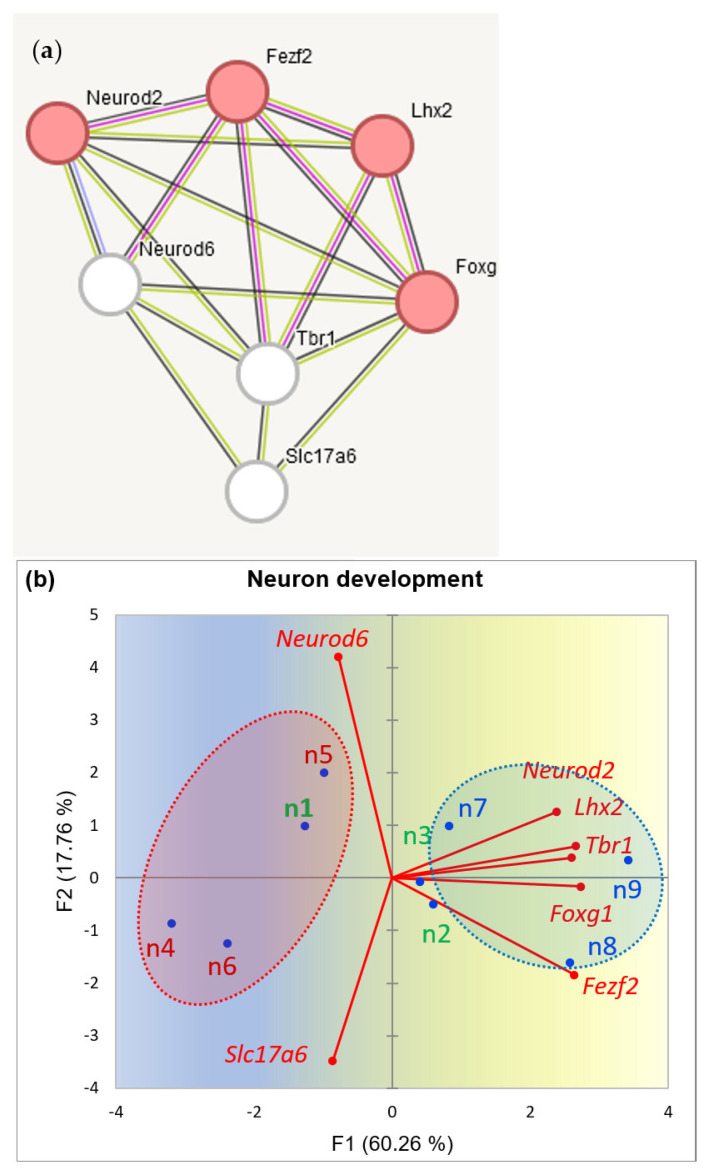
(**a**) HPC Neuron development pathway of BRSGs (red shaded circles; GO: 0048666) and associated ‘multi-purpose’ genes (white circles). (**b**) PCA plot of 9 samples (red-typed—winners, blue—losers; green—control). Random clustering of winners on the left side (red shaded) versus losers (blue shaded) on the right side, rejected with *p*-value < 0.02 (binomial test).

It was reported that increased glutamatergic transmission connected with aggressive bursts has been observed in ventral HPC in isolated post-weaning social isolation mice (Chang et al., 2019) [32], as well as in other reports. We ascertained the social model groups’ trend for neurogenesis BRSGs and plotted the PCA projection, shown in Figure 13.

Figure 13 distinctly outlines opposite trends in neuron development rate between aggressive and depressive groups. Notably, two genes (*Foxg1*, *Lhx1*) maintain certain expression in striatum, while other BRSgs in Figure 13 are highly HPC-specific (Table S1).

We should stress, though, that the effect displayed in Figure 13b might refer specifically to our chronic aggression model of competitive addictive type (see the methods) differed from the spontaneous non-targeted one apparently mentioned in [32].

#### 2.3.8. MRN and VTA GO Annotation

While assessing these brain regions, we encountered an extended shared list of BRSGs (Table 2) compared to three other brain regions considered.

The two midbrain regions considered are the sources of excitatory (dopamine) and inhibitory (serotonin) monoamines. Regions proved to be similar by BRS gene profiles (Table 5 and Table S1); from Table 5, it becomes clear that MRN/VTA BRSGs grossly overlap in their region-specific genes, implying similar neural/synapse architecture. Putting aside *Dbh* (Dopamine Beta-Hydroxylase) BRS gene inherent to VTA neurons, we see glycinergic (*Glra1*, *Slc6a5*,*Slc6a9*) and neurofilament (*Nefh*, *Nefl*, *Nefm*) activities in both regions associated with synapse, kinesin genes, and MAP kinases. 

**Figure 14:**
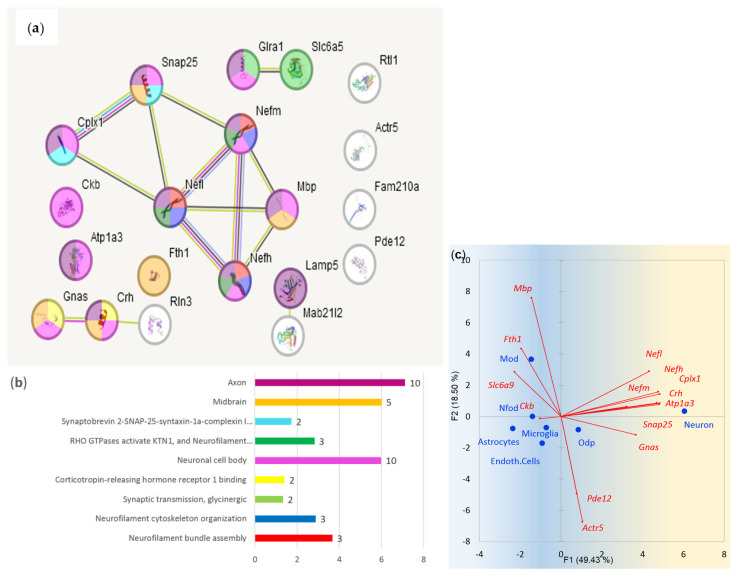
(**a**) 29 MRN/VTA BRS genes’ functional architecture (Table 5). Observed number of edges: 23; expected number of edges: 2. Observed/expected edges ratio yields *p*-value = 1 × 10^−16^ (taken from string-db.org report). (**b**) Sampled GO annotation of 29 MRN/VTA BRS genes. The color scheme corresponds to (**a**). Number of genes per category is attached as bar labels. Full GO annotation is located in Table S6. (**c**) Cell line specific BRSGs distribution based on data in [3] for 7 basic brain cell types. ‘Mod’ abbreviates ‘Myelination oligodendrocytes’; ‘Nfod’ stands for ‘Newly formed olygodendrocytes’, ‘Odp’ denotes ‘Olygodendrocyte precursors’. BRS genes with small expression rates (FPKM < 1) in [3], *Rtl1*, *Mab21l2*, *Slc6a5*, *Glra1*, and *Rln3* (Figure 14a), were dismissed on the plot (**c**).

Based on the GO annotation depicted in Figure 14, we will elaborate on several specific basic processes observed in VTA/MRN regions below by adding up relevant non-BRSG genes, extracted by means of the string-db.org database, according to the corresponding GO term, with the aim of enhancing the confidence in the network expression gradient.

#### 2.3.9. Cellular Matrix Enhancement in VTA/MRN Axons

Figure 14a–c underlines high axonal anterograde and retrograde traffic and transmission activity outlined by genes within midbrain neurons. *Snap25/Cplx1* (Synaptosomal-associated protein 25/complexin 1) pair augmented in dopaminergic/serotonergic neurons provides midbrain-specific monoamine exocytosis as a membrane SNARE complex subunit (Figure 14b) [33]. Glia (oligodendrocytes)-secreted *Fth1* (ferritin heavy chain) is mentioned providing an antioxidant defense system for neurons against iron-mediated cytotoxicity, especially in axon terminals [34]. It is also a member of the autolysosome complex (*Fth1*, *Ftl1*, *Ncoa1*; GO:0044754) [35]. Both regions maintain corticosteroid signaling genes *Crh* and *Gnas*. Considering the expression rate among the gene regions, the glycine metabolism featured by *Glra-Slc6a5-Slc6a9* trio is outstanding (Table 5; Figure 14), exhibiting glycine turnover both by neuronal and glial cells [36,37], featuring intense signaling/metabolic rate specifically in monoaminergic regions. 

#### 2.3.10. Neurofilament Enhancement in VTA/MRN Neurons

**Figure 15:**
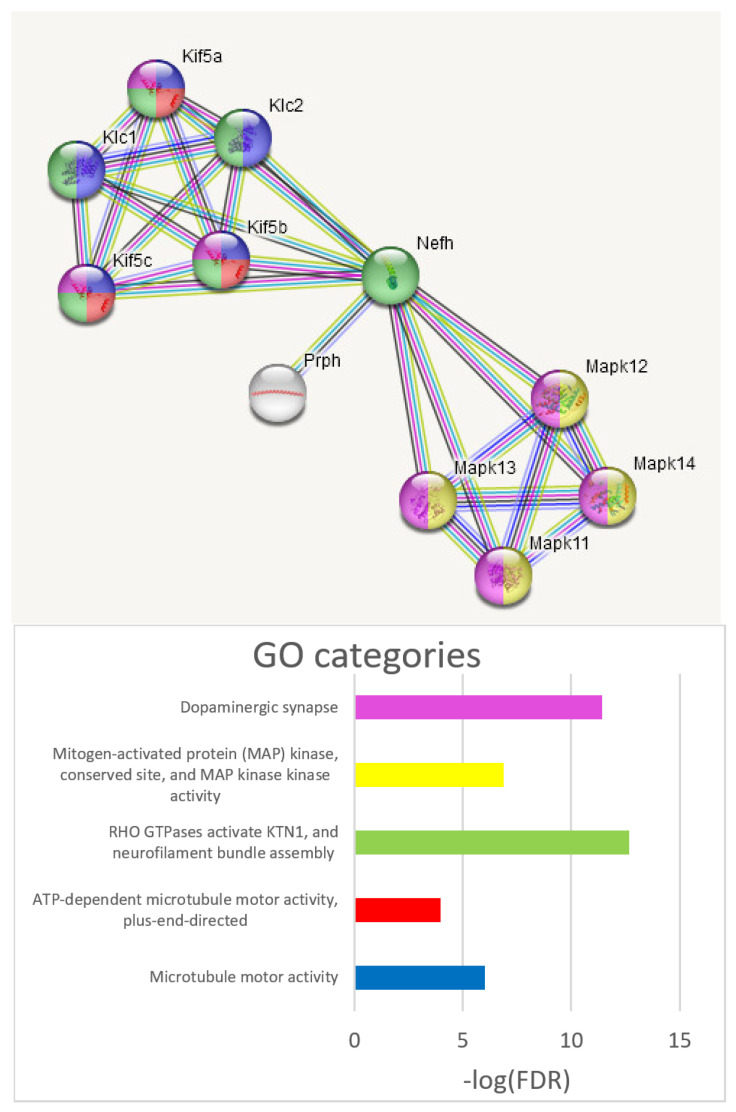
*Nefh* axon-associated complex mediated with “kinesin motor” anterograde system (created by string-db.org by *Nefh* seed). *Nefh* also mediates dopaminergic synapse MAP-kinase network.

Compliant with cell matrix enhancement noted above, both brain regions feature similar axon/synaptic genes expression profile, such as the neurofilament genes (*Nefl*, *Nefh*, *Nefm*; Table 5; Figure 14) and the *Mapk11-Mapk14* kinases characteristic of the dopaminergic synapse, implying intense anterograde axonal transport, as reported earlier. We present the dopaminergic synapse along with kinesin motors in Figure 15.

**Figure 16:**
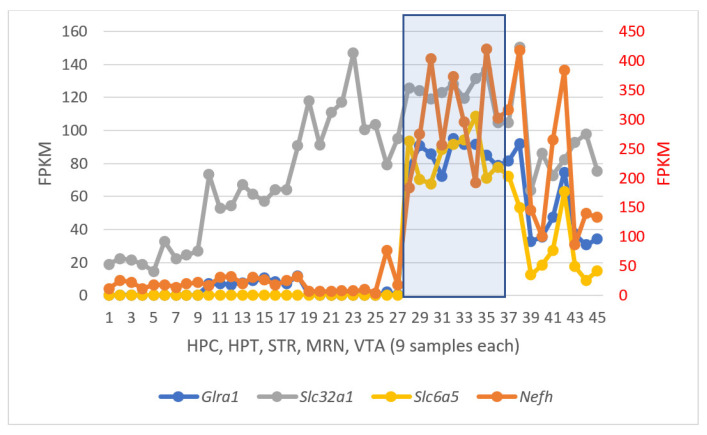
Elevated rate of anterograde axon transport and glycinergic activity across the brain regions based on four BRS genes. MRN region is blue shaded and maintained the highest average gene expression rates (Table 5), while VTA rates (last nine samples) are lower on average.

To illustrate the rate of anterograde transport and glycinergic elevation across the five regions, we have presented the diagram in Figure 16 featuring three glycine-mediated genes and *Nefh* as a neurofilament master gene.

As it seen from Figure 16, the vesicular glycine transport activity (*Slc32a1*) is observed through all brain regions with variable intensity, and is known to be connected with the *NMDA* receptors’ implications [38], while *Glra1*, *Slc6a5*, *Slc6a9*, and *Nefh* are distinctly elevated specifically in monoaminergic regions (Figure 16; samples 28–45), rendering their possible utility as therapeutic targets [38].

Concerning anterograde outstanding rates in VTA/MRN: it is worth mentioning, though, that while the matrix enhancement in VTA/MRN axons makes them high-throughput capable, there is no same elevation rate in retrograde system enhancement, as we observed (not BRSG assigned dyneins gene family, hence not shown). It makes the system prone for retrograde transport rate being ‘jammed’ by the unprecedented anterograde turnover, leading to possible failure of dysfunctional mitochondria cleanup by the unbalanced rates [39,40,41,42].

#### 2.3.11. Myelin Sheath Enhancement in VTA/MRN Neurons

Another point worth mentioning is the VTA/MRN-specific *Mbp* BRS gene (Myelin basic protein). It maintains expression more than 4000 FPKM both in ref. [3] and our data (Table 5). The myelin expression rate is four-fold higher in VTA/MRN regions than in HPT and HPC, while STR manifests a higher expression rate (Table 5), implying a high conductance rate specifically in monoaminergic axons.

**Figure 17:**
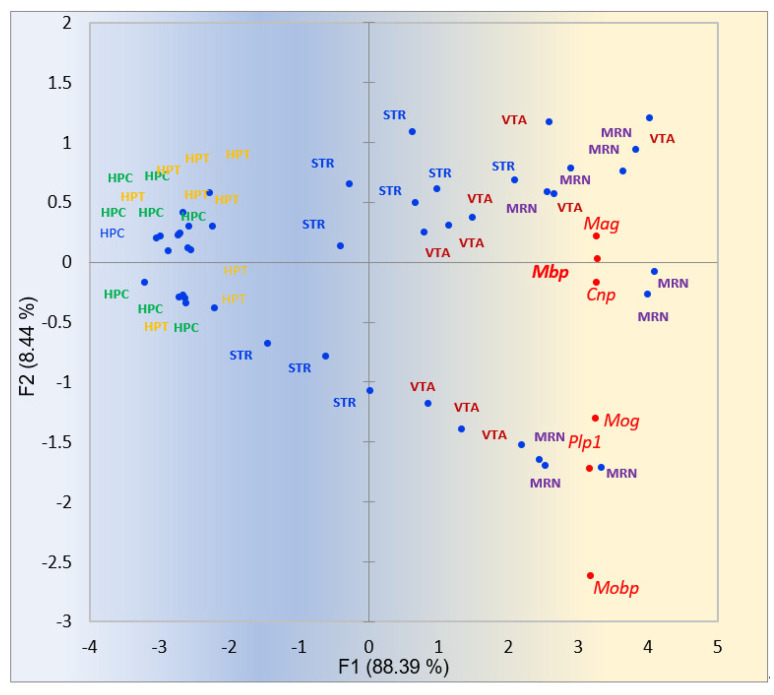
PCA plot of myelin sheath genes activity (GO:0043209; 5 genes associated with *Mbp*) recovered by the *Mbp* seed in string-db.org profiled against 45 samples of 5 brain regions expressed the most in VTA/MRN areas. Green shaded labels are HPC samples, orange shaded ones are HPT, blue colored are STR, red colored are VTA, and purple shaded are MRN. Gradient color shading underlines the elevation of gene expression rate in the right part. Nonrandom clustering of 18 VTA/MRN samples in the right part of the plot is rejected with *p*-value = 3.8 × 10^−6^.

We assessed the Myelin sheath genes network projection (GO:0043209; 7 genes) recovered using the *Mbp* seed in string-db.org profiled in our 45 samples of 5 regions, shown in Figure 17.

#### 2.3.12. Note on Autoreceptor Regulation in VTA/MRN Cells and Implication of Glial Cells

It has been shown that neurons in both VTA/MRN regions maintain autoreceptors coupled to chloride channels [33,43,44]. In the VTA region, autoregulation of the emission of neurotransmitters is observed based on their reuptake and transformation in astrocytes mediated by DRD2 on the pre-synaptic membrane. Based on the concentration of captured neurotransmitters, as well as the state of the extracellular status, astrocytes reduce or increase the release of agents in dopaminergic neurons [45,46]

The MRN maintains an autoreceptor HTR1A, which is highly dense on axons (presynapses) for serotonin reuptake and consequent signaling for increase/decrease of the serotonin synthesis and its release. Having a strong similarity to the VTA in neuron structure and gene expression profile, the MRN, also located in the tegmental region of the midbrain, probably has an astrocyte-mediated serotonergic neuronal firing system similar to the VTA, including the glutamate chain (GLAST/GLT-1 transporters) → GABA interneurons → GABA release → suppression of serotonergic neurons [44].

Such an astrocyte-dependent pattern of monoamines induction explains, firstly, why there are no region-specific neuronal genes (due to the similarity in the structure of neurons used mainly as the monoamine injectors according to the external/internal signal), as well as the strong mitochondrial activity of astrocytes in these cells observed in our data, featured below.

Both regions also maintain extrasynaptic monoamines released majorly from glial cells [45,47].

#### 2.3.13. Increased Mitochondrial Activity in VTA/MRN Regions

**Figure 18:**
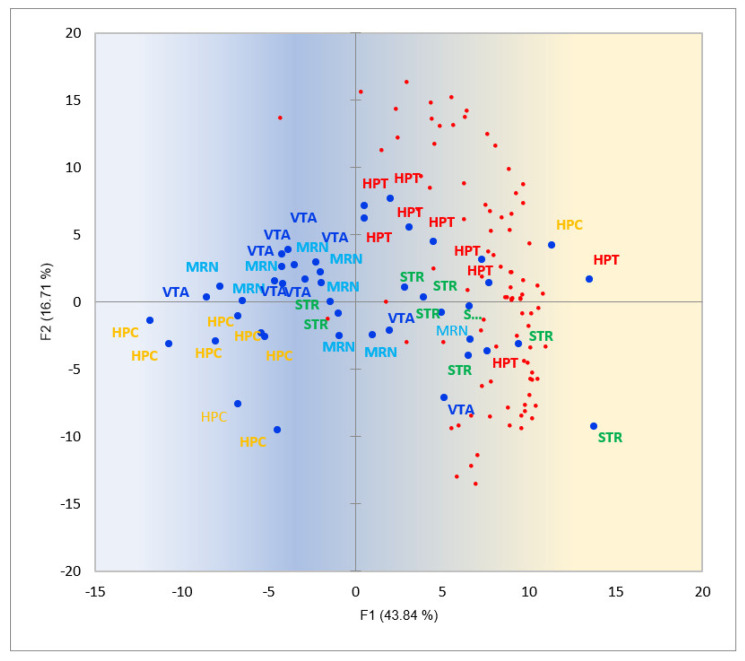
PCA plot of small and large (Rps*/Rpl*) nuclear ribosomal subunits (64 genes; red dots) according to their expression gradients against 45 brain region samples. Blue dots correspond to brain region samples. Gradient coloring underlines gene expression activity direction (blue → yellow). Random clustering of HPT (red labeled) and STR (green labeled) samples on the right side rejected with *p*-value < 7.25 × 10^−5^ (binomial test).

**Figure 19:**
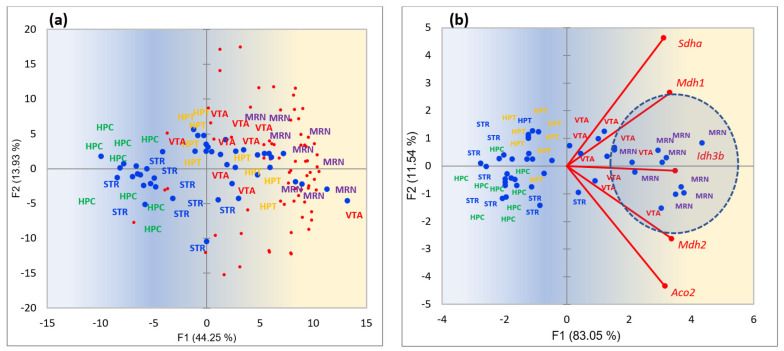
(**a**) Increased activity of mitochondrial ribosome subunit genes (Mrpl*/Mrps*) in the VTA and MRN regions (right half of the graph); (**b**) TCA cycle (*Aco2*, *Mdh1*, *Mdh2*, *Sdha*, *Idh3b*) gene expression rates across five brain regions. Red dots correspond to ribosomal subunit genes projection, blue dots signify the corresponding samples of brain regions. Gradient coloring underlines gene expression activity direction (blue → yellow). The MRN area encircled by a blue shaded oval underscores greater metabolic intensity than VTA. Random clustering of VTA and MRN samples in the right part rejected with *p*-values: (**a**) 6.9 × 10^−5^; (**b**) 3.8 × 10^−6^ (binomial test).

Based on Section 2.3.8. analysis and observations of some mitochondrion-related genes (e.g., *Fam210a*), we decided to inspect the metabolic/energetic activities in the regions by performing comparative analysis of mitochondrial/nuclear ribosomal subunits to get an idea of how regions relate in terms of metabolic rates. For that, we performed PCA analysis on nuclear/mitochondrial ribosomal subunits’ gene expression profiles (Figure 18 and Figure 19).

From Figure 18, we conclude that the STR (seven samples from nine) and HPT (nine samples from nine) regions feature the highest activity in the proteins synthesis while being located at the right half of the plot.

We performed the same analysis for mitochondrial ribosomal subunits (Mrpl*/Mrps*) presented in Figure 19a, and PCA projection of Tricarboxylic cycle (TCA; mitochondrial ATP synthesis) represented by five major TCA enzymes: *Aco2*, *Mdh1*, *Mdh2*, *Sdha*, and *Idh3b* (Figure 19b).

Figure 19 unveils that both mitochondrial ribosome (a) and ATP synthesis (b) activity occurs mostly in the MRN and VTA regions due to the intense synthesis of dopamine and serotonin in neurons and transporting/emitting them to the axon terminals, energetically accommodated by astrocytes [48,49].

#### 2.3.14. Performance of MRN/VTA in Social Conflict Model Groups Assessed Based on the BRSGs

Lastly, we checked out how 18 samples of social stress model behaved in VTA/MRN regions projected on their common BRS genes (Figure 14).

**Figure 20:**
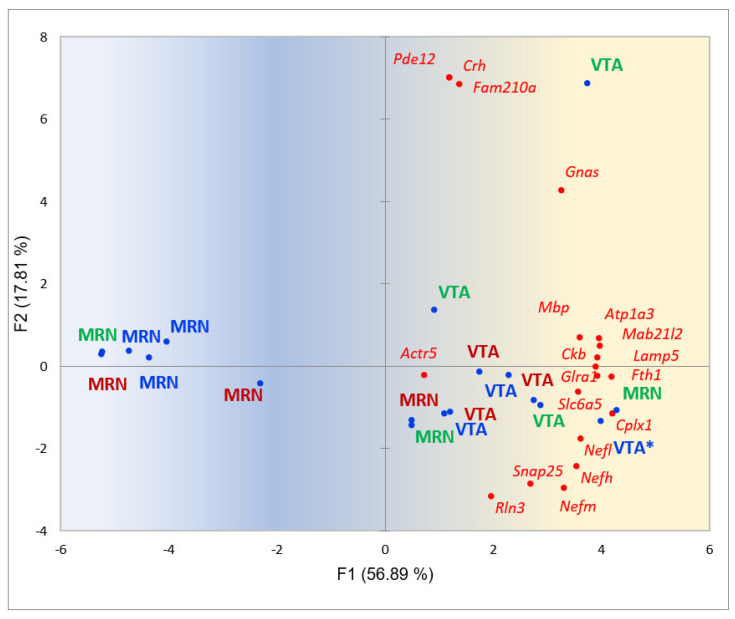
PCA projection of 18 samples (MRN, VTA) against the common pool of BRS genes (Table 5). Green shaded are controls, red labeled are aggressive mice, blue labeled are losers. Blue dots are samples, red dots are BRSGs. Gradient coloring represents BRSG activity trend. *—labeled VTA sample maintains the highest depressive phenotype among looser group.

Figure 20 underscores that midbrain raphe nuclei attenuates serotonin transmission both in losers (blue shaded) and aggressors (red labeled), as previously reported in a range of papers on the depression treatment. Accordingly, it was long established that depressive individuals lack dopamine in STR/Nacc regions [13,50]. We observed herein, that VTA in loser mice synthesizes enough of dopamine proportionally to the severity of depression state. In particular, the blue-shaded VTA label with an asterisk in Figure 20 (right bottom quadrant) manifests the mouse with the most severe case of depression score judging by dorsal striatum state (Figure 5: n26) [13], yielding the high dopamine synthesis outcome in its VTA (according to gene expression rate) across all groups, as we observed therein. We speculate that a lack of dopamine receptors at postsynaptic membranes in MSNs in this mouse preclude taking it, possibly due to striatum glutamate influx lockup [13]. The deficit of serotonin observed in the loser group may also impact/initiate this state, but on a minor scale.

## 3. Discussion

### 3.1. BRSGs Manifest Scaffold of Connected Genes Network in Brain Regions

Herein, we report a nonrandom excess of the number of expected protein–protein interactions in all brain regions’ BRSG sets, implying that the specific functions of the considered regions are carried out by the indicated networks with genes of high activity and regularly specific to a brain region.

In particular, we see specific synaptic genes pathways throughout the three basic brain regions along with other ones: in the hypothalamus, these are hormonal and opioid systems; in the striatum, the genes are of the cAMP signaling pathway; and in the hippocampus, they are glutamatergic system genes. For VTA/MRN regions, the neuronal genes manifest motor genes/matrix genes in the axon anterograde transporting system increase, as does the high energy/catecholamine metabolism.

Note that, due to the specificity of the selection (discriminant genes), most (74%) of the considered BRS genes are neuron-specific ones, since glial genes and their expression profile are fairly similar across brain regions. Because of this, the glia-oriented regions of the VTA, MRN, where the activity of astrocytes is crucial, do not maintain pronounced compartment-specific neuronal genes, except ones maintaining elevated intensity of the axon matrix transport.

### 3.2. Midbrain Monoaminergic Regions VTA, MRN Manifest Common BRS Gene Networks

While few genes involved in Dopamine and Serotonin synthesis were found specific both in VTA (*Dbh*; Table 5 and Table S1) and MRN (*Crh*, *Pde12*, *Actr5*, *Fam210a*, *Rtl1*), the majority BRSG pool is similar (Table 5). The expression rates of *DDS* (L-dopa; nonspecific) and *Tph2* (Tryptophan hydroxylase; VTA/MRN region specific) were approximately equal in both regions, while slightly higher in VTA.

Finally, we outline the major BRSG findings for each region below.

(1) STR: Dorsal Striatum is the region most abundant with BRSGs (Table 1). As 95% of STR neurons comprise Medium Spiny Neurons (MSN), Dopaminoceptive cAMP-mediated pathway is profoundly outstanding in this region by overall expression rate of more than 20 BRS genes (GO: MMU-372790: ‘Gpcr signaling’; Table S2; Figure 3). The ‘motor’ of the cAMP cycle are four phosphodiesterases *Pde10a*, *Pde2a*, *Pde7b*, and *Pde1b*, exemplifying Purine catabolic process (GO:0004115), and are hardly unique for STR, since all regions considered inherently maintain *Gpcrs* and hence evoke c/GMP/cAMP signaling. Still, its STR-specific performance rate is nearly an order of magnitude higher than in any other regions. This activity modulates almost all other pathways, as was shown in ref. [17] and Table S3 (AHC clustering). BRSGs also feature glutamate/dopaminceptive synapses in MSN.

We also correspond BRSG STR-specific transcription factors (TFs) being the members of the specific pathways (Figure 4) pointing at region specific transcriptional regulator genes along with deacetylase activity.

(2) HPT is the most evolutionary ancient region with hormonal/neuropeptide activity featuring the hypothalamic–pituitary–adrenal (HPA) axis for tackling stress response. Thus, neuropeptide/hormonal activity is its major BRSGs pathway (Figure 8). There are also HPT Gabaergic signaling pathway BRSGs (Figure 9, Table 4), and some region-specific transcriptional factors. We also report BRSG makers of arcuate nucleus neuroendocrine neurons (*Ghrh*, *Kiss1*, *Pomc*), paraventricular nucleus neurons (*Oxt*,*Hcrt*,*Pomc*), as well as histaminergic neurons (*Hdc*, *Hcrt*).
(3) HPC is depleted in the density of edges due to its high functional and neuronal heterogeneity (see also Table S7). We may outline only the distinct Glutamatergic signaling pathway, implying a high share of glutamatergic neurons (12 from 21 neuron projection BRSGs), and neuron development genes (*Neurod2*, *Fezf2*, *Lhx2*, *Foxg1*), which proved to be specific for the HPC region.
(4) Besides monoamine-synthesis-specific BRSGs (*Dbh*, *Tph2*), VTA/MRN regions feature enhanced axonal structure BRSGs due to heavy emission and reuptake of monoamines, accommodated by expanding its diameter given increased retro/anterograde transport along with its enhanced myelination.
(5) Some of BRSGs manifest transcription/chromatin modification factors specific for brain regions while employed in the common gene pathways, implying their specific role in the corresponding process relative to the brain region it belongs to.

### 3.3. Application of BRSGs Set in Social Conflict Animal Model: Serotonin Hypothesis of Depression

The serotonin/monoamine deficiency depression hypothesis, outlined in 1963 and further elaborated upon [51,52,53], led to the development of selective serotonin reuptake inhibitors (SSRIs), a class of antidepressants used in the treatment of major depressive and anxiety disorders. However, despite the theoretical basis of this hypothesis, there is currently no direct empirical support for it, as evidenced by a range of recent psychiatric-related journals [52].

In our study, we observed that the use of BRS genes in case–control analysis reduced the background noise generated by the employment of many glial and neuronal Differentially Expressed Genes’ (DEGs) secondary pathways. While DEGs may certainly provide explicit details in many instances, the use of BRS genes allows us effectively and specifically outline the major gene expression features in the groups within each brain region. This is particularly relevant for the Hippocampus (HPC) region, which includes multiple complex heterogeneous gene pathways, including neuron development and others. Initially, we were unable to observe groups clustering while using the entire HPC DEGs’ body due to encountering multiple overlapping events resulting in a gross background noise. BRS genes help us distinctly outline social-group-specific neuronal development trends, shown in Figure 12.

By leveraging Brain Region Specific Genes (BRS genes) in the Ventral Tegmental Area/Medial Raphe Nucleus (VTA/MRN), we were able to examine the effects of these brain regions on chronic social stress using a mouse animal model [11]. This approach is illustrated in Figure 15 of our study. Our findings clearly indicate a reduction in serotonin transmission rates in the MRN of depressive mice. However, as stated in [50], there are some reliable abnormalities in serotonin mechanisms in depressed patients, but their potential role in causing the illness remains to be determined.

Instead of attributing the abrogation of serotonin as a causal factor of depression, we highlight that the release of glutamate in the hippocampus is significantly increased in loser mice compared to other groups (Figure 15) [54,55]. Interestingly, the glutamate expression rate in the hippocampus appears to correlate with dopamine expression in the VTA of loser mice, as well as with the endogenous elevated expression of glutamate in the Stratum (STR) (Figure 1) [13]. This correlation may be mediated by astrocyte emission [56].

Our observation of increased glutamate levels in the STR may explain why loser mice are less likely to uptake dopamine, presumably by blocking dopamine receptor D1 activity [57]. This blockage might occur due to the involvement of STEP/Psd95, which modulates the abundance of competitive NMDA receptors in D1 neurons [57,58,59,60,61,62].

Notably, the *Prkcd* kinase gene expression outburst observed in the STR was previously reported to be closely connected with fear manifestation [20]. *Prkcd* overexpression is accompanied by a profound attenuation of the dopamine-mediated cAMP cycle. We observed this effect in two more loser (depressive) samples in our later data (Figure 7b) [17], confirming this phenomenon as a regular occurrence in depressive states.

Finally, the metabolic networks analysis revealed extensive mitochondrial turnover burden specifically in VTA/MRN regions. The blocking of STR dopamine receptors in depressive individuals is exacerbated by a high dopamine recycling/oxidation rate in the corresponding VTA region, which could potentially lead to oxidative stress, as discussed earlier [63], resulting in subsequent mitochondrial and lysosomal dysfunction, similar to what is seen in Parkinson’s disease.

### 3.4. Limitations of the Study

#### Restricted Brain Region Set

BRSGs were ascertained within five regions considered, but there are many more; thus, some genes may be not uniquely BRSG ones across the whole brain regions set. Still, as we randomly checked our list, and the vast majority are rather BRSG specific-brain-regions wide.

Thus, the BRSGs we used are strictly referred to the five regions considered herein, nevertheless providing a robust-enough scaffold to have an opportunity to compare groups of samples using the ‘major function’ genes within our study.

Another point is that the brain regions considered may contain certain sub-regions/nuclei. For example, HPT region contains Arcuate nucleus featuring opioidergic neurons (*Pomc*-expressing neurons), further split into *Lepr* (leptin receptor), and glucagon-like peptide 1 receptor (*Glp1r*)-expressing neurons, as was recently shown in [64], while histaminergic (monoaminergic) neurons are present specifically in the Tuberomammillary nucleus of the hypothalamus (TMN) [65]. Further study is needed to address these issues.

The animal model employed maintains only male-specific modeling, since the female subjects proved unsuitable for the same protocol due to the lack of challenging (confrontation) factors/instincts between female mice. Consequently, the hormone response, as well as other pathways, may differ in the affected female species while projecting the model on humans.

## 4. Materials and Methods

### 4.1. Samples

We used 45 RNA-Sec mouse tissue samples from 5 mouse brain regions partially employed in [13] the Hippocampus (HPC), Hypothalamus (HPT), midbrain raphe nucleus (MSN), and Ventral tegmental area (VTA) featured in models of chronic social stress studies. Each sample represented a transcription profile of roughly 25,000 genes (transcriptomes) per brain region. Each brain region was represented by 9 samples and 3 groups: control, aggressive, and depressive mice, 3 samples each. No technical controls were involved.

### 4.2. Experimental Animals

Adult C57BL/6 male mice were purchased from Animal Breeding Facility, Branch of Institute of Bioorganic Chemistry of the RAS (ABF BIBCh, RAS) (Pushchino, Moscow region). The housing of animals conformed the standard conditions, namely: 12:12 h light:dark regime starting at 8:00 a.m., at a constant temperature of 22  ±  2 °C. The food was in pellets and the water were available ad libitum. Mice were weaned at 3 weeks of age and housed in groups of 8–10 per standard plastic cage. The age of animals at the time of experiment engagement was 10–12 weeks old.

### 4.3. Ethical Statement

All procedures were in compliance with the European Communities Council Directive 210/63/EU of 22 September 2010. The protocol for the studies was approved by Scientific Council No 9 of the Institute of Cytology and Genetics SD RAS of 24 March 2010, N 613 (Novosibirsk, https://spf.bionet.nsc.ru/, accessed on 1 January 2024).

### 4.4. Experimental Procedures

#### 4.4.1. Protocol for Alternative Social Experiences under Daily Agonistic Interactions in Male Mice

Prolonged negative and positive social experience, social defeats and wins, in male mice were induced by daily agonistic interactions [11,12]. Pairs of weight-matched animals were each placed in a steel cage (14 × 28 × 10 cm) bisected by a perforated transparent partition allowing the animals to see, hear, and smell each other, but preventing physical contact. Before being exposed to encounters, the animals were left at rest for two or three days adapting to new housing conditions and sensory contact. Every afternoon (14:00–17:00 p.m. local time), the cage lid was replaced by a diaphanous one, and after 5 min (the period necessary for individuals’ activation) the cage partition between individuals was removed for 10 min, enabling agonistic interactions. The winning mouse was unambiguously established after two or three physical interactions with the opponent. In particular, the superior mouse would be attacking, biting, and chasing opponent, who displayed only defensive behavior (sideways postures, upright postures, withdrawal, lying on the back, or freezing). Aggressive interaction sessions between males were discontinued by installing the cage partition if the sustained attacks had lasted more than 3 min (in some cases less), preserving the defeated mouse from further attacks. Each defeated mouse (loser) was exposed to the same winner for three days. After the fight session, each loser was placed in an alien cage with an (unfamiliar) winner behind the partition until the next day encounter. On the contrary, the winners were constantly hosted within their original cage. The listed encounter protocol was performed once a day for 20 consequent days. An equal number of the winners and losers were enucleated. The explicit behavioral data on the model has been published in [12].

According to the protocol listed above, we employed three groups of animals in the study: (1) controls—the mice without a consecutive experience of agonistic interactions; (2) losers—chronically defeated mice; (3) winners—chronically aggressive mice. The losers and winners with the most eminent behavioral phenotypes were selected for the transcriptome analysis. Each group comprised three animals in the current study. The winners manifested the highest attacking instances number as well as total attacking time and the shortest latency of the first attack. Aggressive grooming, threats (tail rattling), hostility during 20-day experiment were also manifested. The losers regularly displayed the full submission posture (“on the back”), opponent avoidance and the largest passive defense timespan (freezing, immobility) in the course of agonistic interaction test. Overall, the chronically aggressive mice developed motor hyperactivity, enhanced aggressiveness, and stereotypic-like behaviors, while chronically defeated mice manifested low motor activity and depression-like behaviors. We refer the behavioral data details in our model to be explicitly presented in [11,12].

The control animals and the affected mice were simultaneously decapitated 24 h after the last agonistic interaction. The brain regions were dissected by the same person according to the Allen Mouse Brain Atlas map (https://mouse.brain-map.org/static/atlas accessed on 1 January 2024) [66]. Biological samples were placed in RNAlater solution (Life Technologies, Carlsbad, CA, USA) and stored at −70 °C.

The brain regions selection for the analysis was based on their functions reported elsewhere as implicated in behavior manifestation. They were:

(1) The midbrain raphe nuclei (MRN), a multifunctional region of brain containing the body of the serotonergic neurons;
(2) The ventral tegmental area (VTA) containing the pericaryons of the dopaminergic neurons, which are widely implicated in brain reward circuitry and are important for motivation, cognition, drug addiction, and emotions relating to several psychiatric disorders;
(3) The dorsal striatum (STR), which is a mediator of stereotypical behaviors and motor activity, also implicated in cognitive processes;
(4) The hippocampus (HPC), a part of the limbic system essential for memory consolidation and storage, playing a distinct role in emotional modulation;
(5) The hypothalamus (HPT), which mediates the stress response within Hypothalamic–pituitary axis (HPA), typical for our model.

#### 4.4.2. RNA-Seq Data Collection

We submitted the collected brain samples to JSC Genoanalytica (www.genoanalytica.ru, Moscow, Russia, accessed on 1 January 2024) for RNA-Seq routine. mRNA was extracted using a Dynabeads mRNA Purification Kit (Ambion, Thermo Fisher Scientific, Waltham, MA, USA). cDNA libraries were created using the NEBNext mRNA Library PrepReagent Set for Illumina (New England Biolabs, Ipswich, MA, USA) according to the manufacturer’s protocol. Illumina HiSeq 2500 System (San Diego, CA, USA) was used sequencing using single (non-paired end) reads of 50 bp length. The target coverage was set to 20 Mio. reads per sample. The reads were trimmed using Trimmomatic software, Version 0.39 [67]. The resulting reads were aligned against the GRCm38.p3 reference genome using the STAR aligner, v. 2.7.11a [68]. Cuffnorm software, v. 2.2.1 [69] was employed for expression rate assessment in FPKM units and for the alternatively spliced transcripts’ expression profiles reconstruction. The brain regions were processed for each of the 3 animals per group, separately, without technical replicates. In total, 3 groups of animals were employed in the study, totaling in 9 distinct samples per brain region.

#### 4.4.3. Statistical Methods

We used the XLSTAT-Premium package (xlstat.com, accessed on 1 January 2024); AddinSoft Inc, Paris, France Quote N P201610386) for principal component plotting (PCA) and hierarchical cluster analysis (HLA). The string-db.org (accessed on 1 January 2024) resource was used to annotate gene networks. GO annotation and gene network analysis was also assessed by string-db.org.

#### 4.4.4. Availability of Data and Materials

The additional statistics of data obtained used to support the findings of this study are available from Supplementary Files. The raw sequence data for 45 samples was deposited in ENA archive PRJEB36194.

## 5. Conclusions

We addressed the brain region specific genes (BRSG) as being the attractive pharmacological targets because of their uniqueness in the brain region specific expression. Upon our analysis, we found them highly interconnected within the brain-region-specific gene pathways and responsible for the major brain region functional(s).

We were able to ascertain the specifics of brain region impacts using BRSG methodology for chronic social stress model data. We thus may underscore the utility of our approach using BRSG in similar studies. We are aware that our current approach on BRSG set construction is not exhaustive since there are many other regions that may render other more specific BRSG sets.

## Figures and Tables

**Table 1:** Breakdown of BRSGs based on expression rate.

	Total BRSG	>100 FPKM	>1000 FPKM
VTA	20 *	12 **	6 ***
MRN	22	14	6
HPT	46	13	0
HPC	56	23	3
STR	78	35	3

* 17 BRS genes overlap with MRN (see below). ** 12 BRS genes overlap with MRN; *** 6 BRS genes overlap MRN ones.

**Table 2:** BRSGs pathways’ edges enrichment in each region based on interaction confidence score (string-db.org). The numbers denote #edges/#nodes in the connected network. *p* values were obtained from string-db.org suite.

Interaction Confidence Score (String-db)	STR (78)	HPT (46)	HPC (56)	MRN/VTA (22)	Edges Enrichment *p*-Value
low (>0.150)	838/77	270/43	378/56	67/21	1.00 × 10^−16^
medium (>0.4)	144/52	82/34	59/32	18/14	1.00 × 10^−16^
high (>0.7)	33/28	28/17	17/16 (2.8 × 10^−15^)	9/10	1.00 × 10^−16^
highest (>0.9)	9/13 (3.6 × 10^−6^)	6/7 (1.2 × 10^−8^)	2/4 (0.074)	4/5 (1.6 × 10^−5^)	varies

**Table 3:** GO annotation coding scheme for Figure 4.

BRSGs Seed Pairs	Term ID	Term Description	Gene Count	
***Dlx5–Dlx6***	GOCC:0140368	Decoy receptor complex	3	
Figure 4a	GO:0001649	Osteoblast differentiation	7	
	GO:1901522	**Positive regulation of transcription from RNA polymerase II promoter involved in cellular response to chemical stimulus**	3	
	GO:0006357	Regulation of transcription by RNA polymerase II	9	
***Rarb–Rarg***	GO:0005667	Transcription regulator complex	8	
Figure 4b	GO:0003676	Nucleic acid binding	10	
	CL:4917	**NR1H2 and NR1H3-mediated signaling, and Histone deacetylase 4/5/7/9**	8	
	GO:0048384	**Retinoic acid receptor signaling pathway**	6	
	GO:0035357	Peroxisome-proliferator-activated receptor signaling pathway	3	
	GO:0070562	Regulation of vitamin D receptor signaling pathway	3	
***Egr1–Egr3***	GO:0001228	DNA-binding transcription activator activity, RNA polymerase II-specific	7	
Figure 4c	GO:0140110	**Transcription regulator activity**	9	
	mmu04657	**IL-17 signaling pathway**	5	
	mmu04668	**TNF signaling pathway**	5	
	GO:0002684	Positive regulation of immune system process	5	

**Table 4:** Figure 9 color coding scheme for GO annotation.

BRSGs Seed Pairs	Term ID	Term Description	Gene Count	
***Gabre-Hap1***	GO:0007214	Gamma-aminobutyric acid signaling pathway	4	
Figure 9a	GO:0099536	Synaptic signaling	6	
	GO:0008088	Axo-dendritic transport	4	
***Asb4-Irs4***	GOCC:0070449	Elongin complex	4	
Figure 9b	GO:0031625	Ubiquitin protein ligase binding	5	
	GO:0016567	Protein ubiquitination	9	
***Nnat*-*Peg10***	MP:0003122	Maternal imprinting	4	
Figure 9c	CL:29119	Mostly uncharacterized, incl. COMMD1 N-terminal domain, and Adipokinetic hormone binding	6	

**Table 5:** BRSG subset FPKM values for VTA/MRN brain regions from Table S1. VTA specific genes go first, MRN BRSG next, and MRN/VTA table segment features common genes for the regions. Color coding (string-wise) underlines the expression rate discrepancy between the regions for particular genes, and scales from green to red. Tissue Specific Index (TSI) is at the last column.

	Gene symbol	HPC_avg	HPT_avg	STR_avg	MRN_avg	VTA_avg	TSI
**VTA**	*Tlcd1*	214.57	142.88	20.74	15.01	473.22	0.55
	*Loxl2*	1.03	1.10	1.21	0.86	16.00	0.79
	*Dbh*	0.29	0.05	0.01	3.89	16.42	0.79
**MRN**	*Crh*	0.79	1.99	0.40	**21.17**	4.96	0.72
	*Pde12*	13.72	3.24	16.88	**81.78**	3.52	0.69
	*Actr5*	2.89	3.63	3.58	**97.07**	2.84	0.88
	*Fam210a*	52.14	3.66	3.16	**102.94**	4.00	0.62
	*Rtl1*	2.05	6.46	4.55	**152.14**	3.21	0.90
**MRN/VTA**	***Slc6a5***	0.03	0.04	0.03	84.86	31.97	1.00
	*Mab21l2*	0.09	0.51	0.08	21.92	10.34	0.98
	*Rln3*	0.03	0.26	0.05	29.45	14.85	0.99
	***Glra1***	0.08	8.29	0.36	85.35	51.80	0.94
	*Lamp5*	12.31	6.15	37.59	144.49	53.47	0.78
	*Nefh*	18.10	26.01	16.10	299.94	220.86	0.90
	*Nefl*	206.83	145.48	137.48	820.72	684.99	0.75
	*Nefm*	66.62	57.71	73.11	598.01	507.12	0.85
	*Cplx1*	234.47	113.82	284.92	778.27	555.21	0.68
	*Fth1*	2170.92	1536.80	2882.29	4405.25	3310.33	0.54
	*Atp1a3*	778.55	768.97	592.48	1293.45	1000.71	0.52
	*Mbp*	1033.04	1080.91	2797.41	4590.89	3633.36	0.63
	*Snap25*	715.33	501.60	502.25	1307.61	1046.86	0.58
	*Ckb*	505.70	650.24	1216.06	1329.61	1084.69	0.50
	*Gnas*	634.95	1184.41	315.21	1345.01	1113.15	0.54
	***Slc6a9***	14.11061	50.1118	28.1854	110.2638	88.92087	0.68
	*Tph2*	0.296341	0.084168	0.20817	11.80536	16.09617	0.98

## Data Availability

The RNA-Seq datasets are available in the European Nucleotide Archive (Accession No. PRJEB36194, PRJEB48789).

## Supplementary Materials

The following supporting information can be downloaded at: https://www.mdpi.com/article/10.3390/ijms25052882/s1.

## Abbreviations

BRSG	Brain region specific genes
FPKM	Fragments per kilobase of transcript per million mapped reads
GO	Gene Ontology
KEGG	Kyoto Encyclopedia of Genes and Genomes Pathway Database
PPI	Protein–protein interaction
